# IMMERSIVE DEMENTIA EXPERIENCE: TRANSFORMING DEMENTIA CARE

**DOI:** 10.1093/geroni/igae098.0929

**Published:** 2024-12-31

**Authors:** Kelly O’Malley, Seneca Correa, Julie Boyle, Dawn Stavrakopolous, Kelly Resendes, Olga Quinlan, Amanda Smith

**Affiliations:** VA Boston Healthcare System, Boston, Massachusetts, United States; VA Boston Healthcare System, West Roxbury, Massachusetts, United States; VA Boston Healthcare System, Boston, Massachusetts, United States; VA Boston Healthcare System, Brockton, Massachusetts, United States; VA Boston Healthcare System, Brockton, Massachusetts, United States; VA Boston Healthcare System, Brockton, Massachusetts, United States; VA Boston Healthcare System, Brockton, Massachusetts, United States

## Abstract

The Virtual Dementia Tour (VDT) is an empirically supported training designed to build an understanding of dementia through use of sensory tools and instruction. Trained facilitators guide participants outfitted with devices that alter their senses while they try to complete everyday tasks. The VDT enables participants to experience the physical and mental challenges those with dementia face in order to improve person-centered care. Clinicians (e.g., nurses, nursing assistants, physical and occupational therapists, social workers, psychologists; N = 155) at a Veterans Health Administration facility completed the VDT and provided quantitative and qualitative feedback regarding their satisfaction with the VDT, and the impact this training would have on the care they provide. Participants reported a significant change in their belief that individuals with dementia receive the care they need (83.8% pre, 16.3% post p <.001), indicating that post-training participants felt that individuals with dementia frequently do NOT receive the care they need. The majority (92%) said the experience would change their clinical care practices. Participants strongly agreed that the VDT allowed them to experience what dementia feels like, developed a better understanding of dementia care needs (M =4.66, SD=.51), reinforced or increased their level of knowledge of dementia (M =4.66, SD=.53), and will integrate this in the day-to-day care of person’s with dementia (M = 4.64, SD=.50). Qualitative responses expanded on these findings. The VDT was acceptable to VA direct care staff and is an effective way to build understanding of the needs of older Veterans living with dementia.

